# Supplementary material for: Pandemic-scale phylogenomics reveals the SARS-CoV-2 recombination landscape
Source: Nature. 2022 Aug 11;609(7929):994–7. doi: 10.1038/s41586-022-05189-9 (PMC9519458; doi:10.1038/s41586-022-05189-9)
Supplement: Supplementary file 1 — Supplementary Text 1–18 referenced in the main text, legends for Tables 1–4 and References. [file 41586_2022_5189_MOESM1_ESM.pdf]

nature portfolio

https://doi.org/10.1038/s41586-022-05189-9

Supplementary information

Pandemic-scale phylogenomics reveals the SARS-CoV-2 recombination landscape

In the format provided by the authors and unedited

Supplementary Materials

Supplementary Text

Text S1: Detection of Recombination with Partial Interval Placements
Text S2: Constructing a null model
Text S3: Establishing Significance Under A Null Model Based on Observed Mutation Rates
Text S4: Tree Optimization via Subtree Pruning and Regrafting (SPR) Moves
Text S5: Tree Pruning and Sample Filtration
Text S6: Establishing Sensitivity
Text S7: Filtering Possible False Positives
Text S8: Confirming Variation in Raw Sequence Read Datasets
Text S9: Empirical False Discovery Rate Estimation
Text S10: Measuring Spatial and Temporal Overlap of Recombinant Ancestors using Sample Metadata
Text S11. Genetic Distance Among Donor and Acceptors
Text S12: Permutation Test to Evaluate the Apparent Excess of 3' Recombination
Text S13. Evaluation of Bias and Genetic Distance Between Donors and Acceptors
Text S14: Change-Point Analysis
Text S15. Robustness of The Inferred 3' Bias
Text S16: Estimating R/M
Text S17: Real time detection of recombinant ancestry in newly-sequenced samples
Text S18. Comparison to other recombination detection methods

Tables

Table S1: Acknowledgments table recognizing originating laboratories responsible for obtaining the specimens, as well as the Submitting laboratories where the genome data were generated and shared via GISAID
Table S2: Acknowledgments table recognizing originating and submitting laboratories for data from the China National Center for Bioinformation
Table S3: Acknowledgments table recognizing originating and submitting laboratories for data from the COVID-19 Genomics UK (COG-UK) Consortium [28]
Table S4: Acknowledgments table recognizing originating and submitting laboratories for data from the National Center for Biotechnology Information database [27]

Supplementary Text:

Text S1. Detection of Recombination with Partial Interval Placements
RIPPLES uses the space-efficient data structure of mutation-annotated trees (MATs) [20], in which the branches of the phylogenetic tree are annotated with mutations that have been inferred to have occurred on them, to identify recombination events. Fig. 1 illustrates the underlying algorithm. RIPPLES starts by identifying putative recombinant nodes in the MAT which contain equal or more mutations on its corresponding branch than a user-specified value (the default value is 3). Next, for each putative recombinant node, RIPPLES infers the set of mutations that have occurred on its corresponding sequence by taking into account all mutations annotated on the branches on the path from the node to the phylogenetic root. To determine if this sequence is recombinant, RIPPLES then adds one or two breakpoints on the mutation sites to divide the sequence into two or three segments, respectively. The second segment is referred to as the donor segment, and the first and the third (in case of two breakpoints) segments are referred to as acceptor segments. RIPPLES then uses UShER's [19] highly-optimized and multithreaded phylogenetic placement module to evaluate the parsimony score of partially placing donor and acceptor segments, masking the mutations outside the segment boundaries, on every node of the phylogenetic tree, excluding the nodes that are direct descendants of the recombinant node.

The phylogenetic placement module permits breaking up an internal branch (placing part of the mutations of the branch above the breakpoint and the remaining below the breakpoint) to perform placement on a branch breakpoint if it results in a lower parsimony score. Next, RIPPLES stores all potential donor and acceptor nodes whose partial placement parsimony score is lower than the starting parsimony of the recombinant node. RIPPLES then limits the putative donor and acceptor lists to a maximum of 1000 nodes that provide the largest improvement in the parsimony score during the partial placement. This is to prevent the number of donor-acceptor pairs to become unmanageably large. RIPPLES evaluates each donor-acceptor pair to determine if the parsimony score of partially placing individual segments on the donor and acceptor nodes is lower than the parsimony score of the putative recombinant node by equal or above a user-specified threshold (the default is 3). If found, the putative recombinant node is flagged as a recombinant sequence of the donor and acceptor nodes. RIPPLES also takes into account the mutations on the donor and acceptor nodes to report the maximal genomic intervals within which breakpoint(s) could have occurred without increasing the parsimony score of the partial placements. RIPPLES repeats this process for all recombinant nodes and all possible breakpoint(s) within those nodes.

Because of some rare sub-optimality in tree structure, we sometimes notice that placing the whole putative recombinant node sequence solely on the donor or acceptor sequence can lower the parsimony score relative to the original placement. In such cases, RIPPLES measures the parsimony score improvement of the partial placements relative to the placement which provides the smallest parsimony score for the complete sequence.

Text S2. Constructing a Null Model
It is necessary to define a null model in order to determine whether we observe more recombination events than would be expected as false positives. Here, as an alternative to recombination, we define a null model wherein the additional mutations on a branch that we will test for recombination result instead from the underlying observed mutation process. To do this, we selected nodes at random and added k additional mutations, where k is an input parameter. Here, each mutation was drawn proportionally to the parsimony score of that mutation in the global phylogeny. This should make the extended branches we consider here consistent with the underlying null model. Importantly, our correction for *de novo* mutations should be more appropriate than alternative null

models that assume that the mutation rate is equal across all sites (*e.g., VanInsberghe et al* [17]). Furthermore, to whatever extent recombination contributes to apparently recurrent mutations, this model will be conservative for establishing significance under the null (below).

After generating sequences with additional mutations as described above, we placed those samples onto the phylogeny using UShER [19]. Then, we searched for all possible partial placements using RIPPLES. We record the resulting improvement in parsimony score in the best partial placement that we found relative to the initial placement. The distribution of parsimony score improvement for each initial parsimony score provides a null model for the amount of improvement that might be expected under a model where mutation generates the long branches we search for and conditional on the phylogeny and the initial parsimony score.

Text S3. Establishing Significance Under A Null Model Based on Observed Mutation Rates
For each putative recombinant, we use the null distribution based on mutation on a single phylogeny without recombination to establish significance. For each node with a given initial parsimony score, we obtain the p-value as the proportion of simulated null distribution samples with the same initial parsimony score where the recombinant parsimony score improved by an equal number or more mutations than in the putative recombinant sample. Because the parsimony score improvement distribution is discrete and relatively small in value, the p-values obtained will typically be conservative. Furthermore, our test statistic is defined as the best possible parsimony score improvement for a given set of partial placements for a single node. The number of tests performed should therefore be linear with respect to the number of potential recombinant nodes evaluated. This property will typically be appealing when applying a false discovery rate correction because many tests will be highly correlated among possible parent nodes due to the nodes' proximity within the phylogenetic tree. This can be a problem with methods that are not phylogenetic, *e.g.,* those that examine all possible trios for donor-acceptor-recombinant relationships (e.g., Lam et al. [34]). With such methods, in some cases, if two nodes are distinguished by a SNP that is not contained within a recombinant segment, two or more ancestral nodes can yield identical results. More generally, closely related trios will yield highly correlated results which can impose important challenges for multiple testing corrections.

Text S4. Tree Optimization via Subtree Pruning and Regrafting (SPR) Moves
Optimizing that starting tree for RIPPLES is important for accurately estimating the parsimony score improvement in the partial placements of the recombinant sequence. We found that existing tree optimization tools, such as IQ-Tree [35], do not provide adequate speed and memory efficiencies to handle the massive SARS-CoV-2 phylogenies. Consequently, we developed our own fast and memory-efficient program, called matOptimize, to optimize the parsimony score of the massive SARS-CoV-2 phylogenies. Briefly, matOptimize starts with an input tree and a corresponding VCF file to annotate a set of bases, referred to as the Fitch set, that optimize the total parsimony score of the tree using the Fitch algorithm [36]. All optimizations performed here were done using matOptimize program of commit 2981fcf from https://github.com/yatisht/usher.

Then, matOptimize begins the first optimization round by identifying a set of source nodes which have, or are ancestors to nodes having, recurring or reversal mutations. For each of these source nodes, matOptimize calculates whether a subtree pruning and regrafting (SPR) move for the node within a user-specified radius improves the parsimony score using the incremental update method of Gladstein et al. [37]. matOptimize parallelizes this step across source nodes, assuming all moves are independent. Next, matOptimize identifies which of the profitable SPR moves could conflict (a pair of SPR moves are conflicting if they can affect each other's parsimony score), and applies the non-conflicting profitable moves, prioritizing moves that provide

larger parsimony score improvement among conflicting moves, and re-estimates the Fitch sets and parsimony scores for the affected nodes. matOptimize then starts a new optimization round, but restricts the source nodes to those that were found to improve the parsimony score in the previous round or were within the user-specified radius of the moves that did improve the parsimony score in the previous round. matOptimize keeps performing optimization rounds in this manner until it cannot find any moves that further improve the parsimony score for the entire round.

The matOptimize program is available under the UShER package (https://github.com/yatisht/usher) but further details of this method, as well as relative performance to other methods, will appear in a future publication.

Text S5. Tree Pruning and Sample Filtration

In order to test our method and detect as many SARS-CoV-2 recombination events as possible, we required a large phylogeny encompassing the genetic diversity of the virus. At UCSC, we have been maintaining a daily-updated SARS-CoV-2 phylogeny of all GISAID [26], GenBank [27] and COG-UK [28] sequences using the script https://github.com/ucscGenomeBrowser/kent/blob/master/src/hg/utils/otto/sarscov2phylo/updatePublic.sh and the method described in [19,20]. We started with our phylogeny dated 28/05/2021 containing a total of 1,807,630 sequences with a parsimony score of 1,772,324. We then used the corresponding VCF file and masked all known problematic sites [29] and pruned out samples with fewer than 28,000 non-N nucleotides at positions where the SARS-CoV-2 reference genome had a non-N nucleotide. We also pruned out all samples with 2 or more ambiguous (non-[ACGTN-]) nucleotides, and then iteratively removed all samples on branches with length greater than 30 using the *-b 30* flag in matUtils. After this, we ran matOptimize twice using an SPR radius of 10 and 40 in subsequent rounds, and using the masked VCF as an input. Following this, we again iteratively pruned out all samples on branches with length greater than 30. The final tree contains 1,607,799 samples, 1,967,136 nodes, and has a total parsimony score of 1,522,210.

Text S6. Establishing Sensitivity

To test RIPPLES' sensitivity, we simulated recombinant samples by choosing 2 random internal nodes from our phylogeny with at least 10 descendants and choosing breakpoints at random across the genome. We generated 250 simulations each for one and two breakpoints with 0, 1, 2, and 3 additional mutations added to the sequence after the recombination event. This procedure produced a total of 2,000 simulated recombinants. We ensured that any two breakpoints were at least 1,000 nucleotides apart. The distribution of breakpoints selected for this experiment is approximately uniform, with slight bias against the ends of the chromosomes caused by this 1,000-nucleotide condition.

There are two primary considerations for establishing sensitivity. The first is whether RIPPLES is able to detect a sample as a potential recombinant. Overall, RIPPLES detects 75.8% of all simulated recombinants (77.3% and 74.3% of one- and two- breakpoint recombinants, respectively). If we consider only nodes where the initial parsimony score of the recombinant is placed on a branch of length three mutations or greater (76.3% of simulated samples), the initial requirement to search for recombination in RIPPLES, we detected 90.4% of this subset of recombinants (Extended Data Table 1). Therefore, many potential recombinants, particularly those that inherit relatively little material from either the donor or acceptor, may not be detectable using RIPPLES. Such samples would likely be difficult to detect using other approaches for identifying putative recombinant genomes as well. However, we caution that differences between the nodes we selected to simulate recombination and the true distribution of recombinant nodes might affect our estimated sensitivity (see below). Scripts used to generate simulated recombinants are available at https://github.com/bpt26/recombination/.

Given that RIPPLES detects a sequence as a recombinant, the second consideration is the sensitivity with which RIPPLES identifies an interval containing the true breakpoint position. Among the samples detectable by RIPPLES (i.e. those for which RIPPLES found at least one placement resulting in parsimony score improvement of at least 3), 90% of detected simulated recombinants contain the true breakpoint within one or more of the breakpoint intervals predicted by RIPPLES. 62.5% of the detected simulated recombinants have more than one equally parsimonious breakpoint prediction interval or set of intervals. When RIPPLES is not able to identify a recombination breakpoint prediction interval that contains the true breakpoint position, the identified breakpoint is typically quite close (2010 bp distant on average).

In addition to simulations, we evaluated the sensitivity of RIPPLES by asking if it could detect each of the high-confidence recombinant SARS-CoV-2 clusters of Jackson et al [16]. Briefly, this work used the unique and highly divergent B.1.1.7 haplotype to detect putative recombination events. To do this, we ran RIPPLES while relaxing the requirement that each detected recombinant have a minimum of two descendants. We did this because several of the clusters identified in that work have only a single extant descendant. We found that all putative recombination events identified in that work are also discovered by RIPPLES.

We also simulated recombinants on a much smaller phylogeny in order to test the effect of the phylogenetic inference on detection by RIPPLES. We selected two large clades at random from our dataset (B.1.1.317 and B.1.1.291), and then selected 1,000 random samples from each. We then created 500 recombinants by selecting one random sample from each clade and a random breakpoint. We then inferred a phylogeny *de novo* for each of the simulated recombinants using 2000 samples plus a single recombinant in UShER [19]. In applying RIPPLES to the final phylogenies, we found that our method could detect the proper breakpoint for 410 (82%) of the 500 simulated recombinants when tested in this way, with 72 of the undetected recombinants having a donor and acceptor separated by fewer than three mutations. We therefore conclude that RIPPLES is robust to the challenges of *de novo* phylogenetic inference and if anything, RIPPLES may perform slightly better on smaller phylogenies.

Text S7. Filtering Possible False Positives

We applied several *post hoc* filters to remove putative recombinant nodes that may be false positives resulting from several possible sources of error in consensus genome generation and phylogenetic inference. For each internal node from each trio (putative recombinant, donor, and acceptor nodes) that comprised a recombinant event, we downloaded the consensus genome sequence for the nearest descendants of each node, from COG-UK, GenBank, GISAID, and the China National Center for Bioinformatics. We then aligned the sequences of all descendants for each trio using MAFFT [30], focusing specifically on recombination-informative sites, i.e. where the allele of the recombinant node matched one parent node but not the other. From each set of descendants, we created a consensus sequence for the recombinant, donor, and acceptor nodes. We then compared these consensus sequences to determine whether the informative sites for recombination were likely to be true mutations, or alignment artifacts not captured by our initial VCF file.

If an insertion or a deletion (indel) in the alignment or a set of missing bases (Ns) spanned at least one recombination-informative site in at least one of the consensus sequences, or if an informative site was within 5 nucleotides of an indel or set of missing bases at least 5 nucleotides long, or if more than 5 informative sites were within 2 nucleotides of an indel or set of Ns of any length, we discarded the trio. From careful inspection of individual trios, variation fitting these criteria might be influenced by sequencing quality (e.g. Extended Data

Fig. 3A). We also discarded trios containing more than 5 recombination-informative mutations in a 20-nucleotide span. While multi-nucleotide mutation events do occur, we found upon inspection of the raw sequences that cases of more than 5 mutations in such a small window most often occurred very near to either end of the sequence for that sample (Extended Data Fig. 3B). We then discarded trios where 3 or more recombination-informative mutations in a 20-nucleotide span were found within 5 nucleotides of an indel or set of Ns at least 10 nucleotides in length (Extended Data Fig. 3C). Finally, we removed trios for which the entire set of recombination-informative mutations in the donor or acceptor sequence occurred in a 20-nucleotide span. We have aimed to be conservative with our filtering and excluding these trios may eliminate some true variation from our dataset, but this conservative approach should limit the number of false positives.

To further remove potentially low-quality recombination events, we removed cases whose p-value in 3seq [34] was greater than 0.2. 3seq conducts non-parametric tests for clustering in sequences of binary values. We generated binary sequences using the informative sites for each trio ("A" if the recombinant matched only the donor, "B" if the recombinant matched only the acceptor). Our choice for a p-value of 0.2 is based on visual inspection of binary sequences. For example, a sequence of "AAAABBB" is assigned a p-value of 0.143, and "AAABBB" is assigned a p-value of 0.2. Our intention with this filter is to remove obviously erroneous recombination events, but a recombination event between nodes with few total informative sites could certainly result in such a sequence. However, the sequences "BAAABBABBBBBBA" and "ABBBAABAAAAAAB" result in p-values of 0.275. Clustering in these sequences do not resemble what we expect from simple recombination events and might be the result of contamination or mixed infections.

After controlling for sequence quality, we compared each parsimony improvement to the phylogenetically informed null model described above. We retained only trios whose p-value was less than 0.05, where the p-value represents the proportion of null samples, with parsimony score improvements of at least that observed for the sample of interest, given the same initial parsimony score. We then needed to remove redundant trios from this set of statistically significant predicted recombinants. Several recombinant nodes had predicted recombination events with different sets of parents, and/or different predicted breakpoint intervals, but because multiple recombination events are extremely unlikely to have occurred at one node, we retained only one recombination event for each node. To break ties, we favored recombination events for which we predicted only one breakpoint. Then, we favored trios with fewer informative sites. These represent cases where the donor and acceptor have more similar sequences, and we expect that strains with more similar sequences would be more likely to be in the same place at the same time, as is required for recombination to take place. After this, we resolved the remaining ties by favoring the trio with the smaller 3seq p-value, larger predicted breakpoint interval, and greater sum of descendants of the donor and acceptor nodes. Finally, we found a few cases where two predicted recombinant nodes were the acceptor or donor of each other, and retained only one event for these cases. To accomplish this, we applied the same set of sequential tiebreakers described above. After applying these filters, we retained 589 unique putative recombinant nodes, which are parents to 43,104 unique descendant samples (Data S2). Scripts used for filtering results as described here are available at https://github.com/bpt26/recombination.

Text S8. Confirming Variation in Raw Sequence Read Datasets

To confirm the quality of samples informing the putative recombinant nodes, we manually examined the raw sequence reads for 10 of these samples where we could confidently link the raw sequence read data to a given consensus genome. These raw sequence reads were retrieved from NCBI's SRA database, and were aligned to the Wuhan-Hu-1 reference genome using minimap2 [38]. We then used samtools [39] to convert the output of

minimap2 into manageable bam files, and to create bam index files. The bam files were examined at the informative sites using IGV [40], and they were found to have high consensus (>90%) in each case. This indicates the putative recombinants are unlikely to result from spurious signal due to sample contamination.

Text S9. Empirical False Discovery Rate Estimation
To estimate the false discovery rate associated with our specific approach and statistical threshold selected, we computed a *post hoc* empirical false discovery rate. To do this, we obtained the number of internal nodes that we tested and which were associated with a given parsimony score. Then, for each initial parsimony score and parsimony score improvement, we obtained the expected number of internal nodes that would display that parsimony score improvement under the null model, i.e. as a consequence of mutational processes and in the absence of recombination. We estimate the false discovery rate as the ratio of expected nodes for a given initial and final parsimony score to the number of detected recombinant nodes with the same initial and final parsimony score. As would be expected, more modest parsimony score improvements are associated with a higher estimated false discovery rate (Extended Data Table 3).

Application of a filter to the *post hoc* false discovery rate does not dramatically alter our results. For example, 516 recombinant trios are included in parsimony score improvements that have an FDR ≤ 0.2, and 42,580 unique samples in our tree (2.6%) are descended from these recombinant nodes. Using only these recombinant trios, we detect a changepoint in breakpoint density at genomic coordinate 20,899, and a R/M value of 0.00260.

Text S10. Measuring Spatial and Temporal Overlap of Recombinant Ancestors using Sample Metadata
We performed *post hoc* analysis using sample metadata to determine if the ancestors of the recombinant nodes had higher spatial or temporal overlap than expected by chance, as follows. We treated geography as a categorical variable at the country level. We computed geographic overlap as the joint probability of choosing a sample from the same country from the descendants of the donor and the acceptor nodes. For temporal overlap, we recorded intervals from the earliest to the most recent sample descended from the donor and acceptor, respectively, and calculated the minimum number of days separating the two intervals (with 0 for overlapping intervals).

We generated a null distribution for both categories by selecting, for each detected trio, two random internal nodes from the tree with a number of descendants equal to the real donor and acceptor respectively. We then calculated geographic and temporal overlap in the same way for this random set. This is a conservative null because the most closely related nodes are often the most similar with respect to spatio-temporal metadata and therefore, the hardest to detect for recombination. Additionally, because of uneven sequencing efforts both temporally and geographically, as well as the sometimes ambiguous choice of donor and acceptor (e.g., because a mutation that distinguishes two nodes is not contained in a recombination tract), we would not expect perfect overlap in these metadata categories. It is important to note that we break ties between donor/acceptor pairs entirely without considering descendant metadata which would invalidate this *post hoc* analysis. Nonetheless, as we anticipate for true recombinants, both metadata categories are strongly enriched for similarity among donor and acceptor nodes.

Text S11. Genetic Distance Among Donor and Acceptors
In general co-circulating strains, which are required for the possibility of recombination, are often genetically similar. When we examined the genetic distances among the inferred donor and acceptors for each putative recombinant, we discovered this expected pattern. Specifically, both the initial parsimony score of the sample

placement, and the estimated genetic distance between the donor and acceptor are smaller than what we find when nodes are selected at random as in simulations (Extended Data Fig. 5). This implies that the true false negative rate for RIPPLES' might be larger than it would be if recombinants occurred between random nodes.

Text S12. Permutation Test to Evaluate the Apparent Excess of 3' Recombination
We next sought to determine if identified recombination breakpoints are shifted towards the 3' end of the genome. To do this, we performed a permutation test comparing the difference of the mean of the distribution of detected breakpoints when recombination breakpoints are simulated uniformly at random with the mean of the breakpoint position distribution in the true set. Briefly, this is accomplished by randomizing the set of breakpoint positions between two vectors of equivalent lengths to the simulated and real sets. The reported p-value is the proportion of such permutations where the difference between the mean position of the true and simulated vectors was greater than or equal to the observed difference in the true data. Importantly, because both distributions reflect subsets of recombination events that can be detected conditional on the landscape of genetic diversity and phylogeny of SARS-CoV-2, this is an improved null comparison than assuming a distribution, *e.g.,* a uniform distribution. To control for genetic distance, we also conducted a permutation test drawing only from simulated detected breakpoints whose acceptor and donor were separated by fewer than 10 mutations, and found that our identified breakpoints were still significantly shifted towards the 3' end of the genome ($p = 5\times10^{-6}$).

Text S13. Evaluation of Bias and Genetic Distance Between Donors and Acceptors.
There are at least two plausible differences between randomly selected nodes and uniformly-selected recombination breakpoints that we used to evaluate RIPPLES and the distribution of recombination breakpoints within natural isolates of SARS-CoV-2. First, it is plausible that recombination rates vary across the viral genome. Indeed, characterizing the landscape of recombination in SARS-CoV-2 is one of the analysis goals of this work. Second, the genetic distances among donors and acceptors is likely to differ from a random distribution. In particular, we expect that strains of SARS-CoV-2 that co-circulated within host populations will typically be more genetically similar than randomly selected nodes in the phylogeny. Moreover, it is possible that these two factors are interrelated. Here we evaluate these two possibilities using the simulated datasets described above.

One important consideration is whether RIPPLES' power to detect putative recombinants varies systematically with genetic distance between donor and acceptor nodes. As expected, we find that sensitivity increases with increasing genetic distance among the putative recombinants. *I.e.,* the probability of successfully detecting a simulated recombinant is positively correlated with the genetic distance separating the donor and acceptor ($p < 2.2e-16$, 2.2e-16 for one and two breakpoint recombinants respectively; Wilcoxon rank sum test). Similarly, the initial parsimony score for the placement of the recombinant node, which we expect to be related to the genetic distance among the parents and is directly observable in real data, is also strongly positively correlated with successful detection of simulated recombinants ($p < 2.2e-16$, 2.2e-16 for one and two breakpoint recombinants respectively; Wilcoxon rank sum test).

Finally, if the probability of successfully detecting recombinant nodes varies systematically across the viral genome, this could produce spurious inferences of a non-uniform recombination map even when no such bias exists in reality. This effect might also be compounded by the genetic distances between recombinant nodes. We therefore investigated the relationship between breakpoint position, genetic distance among the parents, and the

probability of successfully detecting recombinations. When we consider all the data in aggregate, we find that the false negative rate is similar across the viral genome, but highest towards the extreme approximately 1000bp 3' and 5' positions in the genome (Extended Data Fig. 1A). This is expected because the signature of recombination requires a consecutive stretch of recombinant ancestry to be detected by our method. In examining the false negative rates associated with the most genetically similar donor and acceptor nodes (the 20% most similar in our simulated set, Extended Data Fig. 1B), we find that there is a very slight bias where the middle portion of the genome (coordinates 10,000 - 19,000) displays a true positive rate of approximately 0.42. Whereas the 3' segment of the genome (coordinates 19,000 - 28,903), displays a slightly lower true positive rate of 0.36. We observe the lowest true positive rate, 0.27, in the 5' segment of the genome (coordinates 1 - 10,000).

Because there is only a minor bias in the estimated true positive rate across the SARS-CoV-2 genome we anticipate that if RIPPLES successfully detects a significant deviation from uniform recombination rates in the real data, it will reflect a true difference in the recombination landscape among detectable recombinant lineages. Furthermore, we note that although most true recombinants likely form between genetically closely related lineages, the bias we discovered is actually opposite of the effect of this detection bias (see below). This makes our test, which assumes no such bias, conservative with respect to the recombination landscape we report here. Nonetheless, we caution that it is likely that true properties of recombination differ from what we have simulated and all interpretation is conditioned on the subset of recombination events that can be detected using our approach.

Text S14. Change-Point Analysis

To identify intervals where the frequency of recombination breakpoint intervals differs along the genome, we performed change-point analysis. We do this using the changepoint R package [31], and fit a Poisson model to the count of recombination prediction interval midpoints. Because we have reduced ability to detect recombination events towards the edges of the SARS-CoV-2 genome, we removed the first and last thousand basepairs of the genome from this analysis (*i.e.,* we considered coordinates 1,000 - 28,903). We then computed the mean rate of recombination breakpoints within the intervals on either side of the identified change-point to estimate the fold increase in recombination rate in the 3' portion of the genome and we assessed significance using a poisson test.

Text S15. Robustness of The Inferred 3' Bias

If errors that generate false recombinants occur disproportionately toward the 3' end of the genome, this might also generate the observed excess of recombination. If so, we would expect that nodes with more descendants should more closely approximate the true distribution of recombination events. When we subset the data to the number of putative recombinant nodes with five or more descendant sequences, we observe a slightly greater bias with a 2.45 fold excess recombination rate in the 3' segment of the genome ($P < 6.7e-15$, Poisson test). This suggests that the rate of discovered putative recombination events is relatively unaffected by the number of descendant samples observed.

We and others have demonstrated that SARS-CoV-2 genomes sometimes contain non-random errors [29,41] which could affect inference of recombination if pervasive. Here we excluded all known positions, however it is impossible to exhaustively confirm all possible recombination informative positions. We have previously shown that country of origin is often a good proxy for originating labs [42], and we therefore sought to determine if similarly biased recombination landscapes are apparent across samples for many geographically diverse origins. To do this, we subdivided the inferred recombination breakpoints by country of origin for five of the most

highly sampled countries in the dataset. We then asked if the rate of recombination interval midpoints is significantly elevated in the 3' portion of the genome using only the subset of putative breakpoints with at least one descendant sample from that country. In each comparison, we found a bias consistent with our inferred breakpoint bias in the full dataset suggesting that our results are robust to biases associated with the originating labs (Extended Data Table 5).

Inter-lineage recombination events that include highly genetically dissimilar donor-acceptor pairs also support a 3' bias in recombination rates. Because the Alpha and Omicron lineages are highly genetically divergent relative to all strains circulating prior to their emergence, it is straightforward to identify inter-lineage recombination events with either highly divergent lineage [16,43–48]. These include Alpha recombinants and Delta-Omicron recombinant lineages. When we examined this set of inferred recombination breakpoints, we found that 8 breakpoints occurred 5' of the change-point that we identified above and 11 breakpoints occurred toward the 3' of the identified changepoint. This implies a relative rate of 3.151 recombination events per bp (P = 0.01269; Poisson test; confidence interval = 1.154–9.023). Although other biases might impact the identification of such recombinants, these data further support our inference of an excess of recombination in the 3' portion of the viral genome.

Text S16. Estimating R/M

A central focus of much of microbial evolutionary analysis is distinguishing the relative contributions of recombination and mutation to patterns of variation. To estimate this ratio for SARS-CoV-2, we conservatively assume that RIPPLES successfully detects all recombination events that are present on the phylogeny. Then, the decrease in parsimony score associated with each detected recombination event is an estimate of the total variation that results from recombination. The contribution to the total mutations present in the viral population is then the parsimony score decrease multiplied by the number of descendant lineages of that recombinant node. This is the total number of observed mutations whose genealogies contain a recombination event. For lineages descendant of multiple recombinant nodes, we multiplied by the recombinant node with greater parsimony score improvement. If we subtract this value from the total number of mutations observed across the entire datasets, we obtain an estimate of the number of mutations whose histories are attributed in whole to mutational processes. The ratio of these two numbers is an estimate of R/M averaged across all samples that are included in our tree.

Text S17. Real time detection of recombinant ancestry in newly-sequenced samples

Tens of thousands of new SARS-CoV-2 sequences are being added to online databases every day [26–28]. In order to facilitate the real time discovery of novel recombination events as well as the presence of known recombinant ancestry in these sequences, RIPPLES includes an option to restrict the search for recombinant nodes to a set of user-specified samples and their ancestors. This allows RIPPLES to be used in conjunction with UShER [19] to incorporate newly-sequenced SARS-CoV-2 samples in the comprehensive global phylogeny and identify among them those with a recombinant ancestry in real time. For example, on a server using 40-core Intel Xeon E7-4870 processor, incorporating 10 new samples in a mutation-annotated tree (MAT) of 1.607 million SARS-CoV-2 sequences takes 26 seconds using UShER, and using the output MAT to identify from the newly-incorporated samples those having a recombinant ancestry takes an additional 8 minutes 34 seconds using RIPPLES, on average. We have added a tutorial for RIPPLES to our wiki, which is available at https://usher-wiki.readthedocs.io/en/latest/tutorials.html.

Text S18. Comparison to other recombination detection methods.

We sought to compare RIPPLES to previously developed recombination detection software, although direct comparisons are made difficult by the fact that RIPPLES requires a mutation-annotated tree while other methods use whole-genome trio sequences. We have compared RIPPLES to RDP [49] in searching for evidence that one particular sample in a 1,000-sample dataset is a recombinant, in order to test the scaling capabilities of RIPPLES. RDP exhaustively tests triplets within a set of whole-genome sequences. Using 224 vCPU, RIPPLES requires less than one second for this search, whereas RDP requires over one hour. While both programs found evidence for recombination in this sample, we concluded that RDP was prohibitively slow for any meaningful accuracy comparison on our full dataset of more than one million total genomes.

We also examined the same 500 recombinant trios assessed earlier by RIPPLES (Text S6), this time specifying the two parents and simulated recombinant child. Here, we compare against the approach 3seq, which has been used in previous analyses of SARS-CoV-2 recombination. Using this approach, 3seq [34] finds the proper breakpoint for 491 of the 500 trios. However, testing in this way would be impossible unless the identity of the recombinant sample and its two parents were already known. The reason is that the multiple testing correction is prohibitively expensive with so many highly correlated tests due to the underlying but unmodelled shared phylogenetic history of the sample [16].

When we use 3seq to examine the entire 1289-sequence dataset (500 simulated recombinants and 1,000 parents each, chosen with replacement), 2.13×10^9 triplets are examined and a p-value of 2.34×10^{-11} is required for rejection of the null hypothesis per the 3seq documentation, and 0 recombinants are detected. We emphasize that the very nature of exhaustive recombination searches via trios fundamentally prevents detection of recombination without prior knowledge of the recombinant sample and its parents due to the need for extensive p-value corrections. This search of 2.13×10^9 triplets also takes roughly 24 hours to complete on a server with 80 processors (Intel Xeon CPU E7-4870 at 2.40GHz), each with 10 CPU cores, and scales at $O(n^3)$, where n is the number of sequences under consideration. Even if 3seq was used on a restricted search of just 47,971 branches of the mutation-annotated tree which contained 3 or more mutations, the number of triplets would still reach 1.1×10^{14}, and the entire search would take roughly 50,000 days on this hardware configuration.

REFERENCES

34. Lam, H. M., Ratmann, O. & Boni, M. F. Improved Algorithmic Complexity for the 3SEQ Recombination Detection Algorithm. *Mol. Biol. Evol.* **35**, 247–251 (2018).
35. Minh, B. Q., Lanfear, R., Trifinopoulos, J., Schrempf, D. & Schmidt, H. A. IQ-TREE version 2.1. 2: Tutorials and Manual Phylogenomic software by maximum likelihood. (2021).
36. Fitch, W. M. Toward Defining the Course of Evolution: Minimum Change for a Specific Tree Topology. *Syst. Biol.* **20**, 406–416 (1971).
37. Gladstein, D. S. Efficient incremental character optimization. *Cladistics* **13**, 21–26 (1997).
38. Li, H. Minimap2: pairwise alignment for nucleotide sequences. *Bioinformatics* **34**, 3094–3100 (2018).
39. Danecek, P. *et al.* Twelve years of SAMtools and BCFtools. *Gigascience* **10**, (2021).
40. Robinson, J. T. *et al.* Integrative genomics viewer. *Nat. Biotechnol.* **29**, 24–26 (2011).
41. De Maio, N. *et al.* Mutation Rates and Selection on Synonymous Mutations in SARS-CoV-2. *Genome Biol. Evol.* **13**, (2021).
42. Gozashti, L. *et al.* Updated analysis with data from 4 March 2021. *Virological* https://virological.org/t/issues-with-sars-cov-2-sequencing-data/473/15 (2021).
43. *Omicron Recombinant Lineage with Likely Breakpoint at NSP3 (30 Seqeuences in the UK as of 2022/02/17) · Issue #445 · cov-lineages/pango-designation*. (Github).
44. *Denmark [~30 seqs, isolated/passaged in Vero] · Issue #444 · cov-lineages/pango-designation*. (Github).
45. *AY.119* recombinant (7 seq) [potentially the one monitored by UKHSA] · Issue #439 · cov-lineages/pango-designation*. (Github).
46. *BA.1 recombinant lineage under monitoring in the UK · Issue #441 · cov-lineages/pango-designation*. (Github).
47. *Small Australian cluster (7 seq) of potential Delta-Omicron recombinants · Issue #437*. (Github).
48. Possible English recombinant descended from Omicron and Delta (UKHSA ‘signal under monitoring’) · Issue #422. https://github.com/cov-lineages/pango-designation/issues/422.
49. Martin, D. P. *et al.* RDP5: a computer program for analyzing recombination in, and removing signals of recombination from, nucleotide sequence datasets. *Virus Evol* **7**, veaa087 (2021).
