# Supplementary material for: Pandemic-scale phylogenomics reveals the SARS-CoV-2 recombination landscape
Source: Nature. 2022 Aug 11;609(7929):994–7. doi: 10.1038/s41586-022-05189-9 (PMC9519458; doi:10.1038/s41586-022-05189-9)
Supplement: Supplementary file 2 — Reporting Summary [file 41586_2022_5189_MOESM2_ESM.pdf]

nature research

Corresponding author(s): Yatish Turakhia
Russell Corbett-Detig

Last updated by author(s): August 18, 2021

Reporting Summary

Nature Research wishes to improve the reproducibility of the work that we publish. This form provides structure for consistency and transparency in reporting. For further information on Nature Research policies, see our Editorial Policies and the Editorial Policy Checklist.

Statistics

For all statistical analyses, confirm that the following items are present in the figure legend, table legend, main text, or Methods section.

n/a	Confirmed	
☒	☐	The exact sample size (*n*) for each experimental group/condition, given as a discrete number and unit of measurement
☐	☒	A statement on whether measurements were taken from distinct samples or whether the same sample was measured repeatedly
☐	☒	The statistical test(s) used AND whether they are one- or two-sided<br>*Only common tests should be described solely by name; describe more complex techniques in the Methods section.*
☒	☐	A description of all covariates tested
☐	☒	A description of any assumptions or corrections, such as tests of normality and adjustment for multiple comparisons
☐	☒	A full description of the statistical parameters including central tendency (e.g. means) or other basic estimates (e.g. regression coefficient) AND variation (e.g. standard deviation) or associated estimates of uncertainty (e.g. confidence intervals)
☐	☒	For null hypothesis testing, the test statistic (e.g. *F*, *t*, *r*) with confidence intervals, effect sizes, degrees of freedom and *P* value noted<br>*Give P values as exact values whenever suitable.*
☒	☐	For Bayesian analysis, information on the choice of priors and Markov chain Monte Carlo settings
☒	☐	For hierarchical and complex designs, identification of the appropriate level for tests and full reporting of outcomes
☒	☐	Estimates of effect sizes (e.g. Cohen's *d*, Pearson's *r*), indicating how they were calculated

Our web collection on statistics for biologists contains articles on many of the points above.

Software and code

Policy information about availability of computer code

Data collection	All data used in this work are available from GISAID (gisaid.org), COG-UK, and Genbank, with specific sample accessions listed in Supplemental Tables 5-8.
Data analysis	The data was analyzed using code available at https://github.com/yatisht/usher and https://github.com/bpt26/recombination. All software versions are indicated where appropriate in the methods section of the manuscript.

For manuscripts utilizing custom algorithms or software that are central to the research but not yet described in published literature, software must be made available to editors and reviewers. We strongly encourage code deposition in a community repository (e.g. GitHub). See the Nature Research guidelines for submitting code & software for further information.

Data

Policy information about availability of data

All manuscripts must include a data availability statement. This statement should provide the following information, where applicable:

- Accession codes, unique identifiers, or web links for publicly available datasets
- A list of figures that have associated raw data
- A description of any restrictions on data availability

All data used in this work are available from GISAID (gisaid.org), COG-UK, and GenBank, with specific sample accessions listed in Supplemental Tables 5-8.

Field-specific reporting

Please select the one below that is the best fit for your research. If you are not sure, read the appropriate sections before making your selection.

☐ Life sciences ☐ Behavioural & social sciences ☒ Ecological, evolutionary & environmental sciences

For a reference copy of the document with all sections, see nature.com/documents/nr-reporting-summary-flat.pdf

Ecological, evolutionary & environmental sciences study design

All studies must disclose on these points even when the disclosure is negative.

Study description	In this study, we describe an efficient method that exhaustively searches a phylogeny with applications demonstrated for the current SARS-CoV-2 global phylogeny. We compared our approach to many existing methods and documented accuracy (on simulated data), consistency (with empirical data), compute time and memory usage requirements.
Research sample	Our study is based on existing dataset of SARS-CoV-2 sequences shared via GISAID (gisaid.org), GenBank, and COG-UK. The specific sample accessions are listed in Supplementary Tables 5-8.
Sampling strategy	Not relevant. We chose to work primarily with our 28/5/21 public release of the SARS-CoV-2 phylogeny, because in order to develop our software, we needed a constant tree to perform experiments on and these were the most up-to-date available at the time we began this work. We also worked with simulated data, designed to behave similarly to the real data, as described in our Methods section.
Data collection	All sequences marked as 'complete' and 'high coverage' submitted up to 28/5/21 were downloaded from GISAID (gisaid.org), as well as sequences from GenBank, and COG-UK, were used to build the global phylogeny after a few additional filtering steps (Methods). These data are from a collection of sequences obtained throughout the world during the SARS-CoV-2 pandemic. Supplementary Tables 5-8 list all individuals responsible for the primary data collection in all sequences used in this study.
Timing and spatial scale	All sequences present in the 28/5/2021 public tree were used, except for those pruned out according to our Methods section. We chose 28/5/21 because we needed a consistent sample with which to hone our methods and conduct experiments, as well as to have a "reference tree" to refer back to throughout the study.
Data exclusions	Incomplete and low-coverage sequences as well as those with known sequence issues were excluded (Methods). Our previous study and other related studies cited in the Methods demonstrate that errors can lead to false nucleotide substitutions for myriad reasons unrelated to the biology of the virus itself. We have masked these sites from our analysis and the specific criteria for exclusion are indicated in the method section.
Reproducibility	All our findings and results are completely reproducible using the code and data available from https://github.com/yatisht/usher. Simulations and filtration of sequences were conducted using code from https://github.com/bpt26/recombination.
Randomization	Not relevant. We used identical dataset for all comparative analysis hence randomization is not necessary for comparing results of the approaches used in this study.
Blinding	Blinding is not relevant because experimenter bias cannot affect the results of this analysis.

Did the study involve field work? ☐ Yes ☒ No

Reporting for specific materials, systems and methods

We require information from authors about some types of materials, experimental systems and methods used in many studies. Here, indicate whether each material, system or method listed is relevant to your study. If you are not sure if a list item applies to your research, read the appropriate section before selecting a response.

Materials & experimental systems

n/a	Involved in the study	
☒	☐	Antibodies
☒	☐	Eukaryotic cell lines
☒	☐	Palaeontology and archaeology
☒	☐	Animals and other organisms
☒	☐	Human research participants
☒	☐	Clinical data
☒	☐	Dual use research of concern

Methods

n/a	Involved in the study	
☒	☐	ChIP-seq
☒	☐	Flow cytometry
☒	☐	MRI-based neuroimaging
